# Distinct lipidomic profiles but similar improvements in aerobic capacity following sprint interval training versus moderate-intensity continuous training in male adolescents

**DOI:** 10.3389/fphys.2025.1475391

**Published:** 2025-01-30

**Authors:** Wantang Su, Jianming Liu, Aozhe Wang, Haifeng Zhang, Yaqi Sun, Zhiyi Yan, Michael Svensson, Ji-Guo Yu, Li Zhao

**Affiliations:** ^1^ Department of Exercise Physiology, Beijing Sport University, Beijing, China; ^2^ School of Competitive Sports, Beijing Sport University, Beijing, China; ^3^ School of Physical Education and Sports Science, Qufu Normal University, Qufu, Shandong, China; ^4^ Beijing Municipal Key Laboratory of Child Development and Nutriomics, Capital Institute of Pediatrics, Beijing, China; ^5^ Department of Community Medicine and Rehabilitation, Section of Sports Medicine, Umeå University, Umeå, Sweden

**Keywords:** lipidomic, aerobic capacity, moderate-intensity continuous training, sprint interval training, adolescents

## Abstract

**Background:**

Exercise-induced metabolic changes, especially lipidomic changes are generally associated with improvements in cardiovascular health. Despite numerous previous studies, the differences in lipidomic profile response to different types of exercise training remain unclear. This study aimed to investigate how two different exercise intensities affect aerobic capacity and serum lipidomic profiles in healthy adolescents.

**Methods:**

Twenty-four healthy untrained male adolescents (13.08 ± 0.88 years old) were recruited and randomly assigned to moderate-intensity continuous training (MICT) group or sprint interval training (SIT) group to complete a specific training on a cycle ergometer for 6 weeks. Peak oxygen uptake (VO_2_peak) and body composition were measured, and blood samples were collected for serum lipoproteins and lipidomic analysis. Anthropometric, VO_2_peak, and serum biochemical data were analyzed using two-way repeated analysis of variance, while targeted lipidomic analysis was performed by principal component analysis and paired-sample *t*-test.

**Results:**

VO_2_peak significantly improved from 39.05 ± 8.17 to 47.52 ± 8.51 [F (1, 44) = 14.75, *p* < 0.05] for MICT and from 40.13 ± 6.37 to 48.42 ± 7.01 [F (1, 44) = 14.75, *p* < 0.05] for SIT. A total of 28 lipids in MICT and 5 lipids in SIT showed significant changes out of 276 identified lipids (FC > 1.5 or <1/1.5, FDR <0.05). In MICT, 21 lipids, including sphingolipid (SP) and phospholipid (PL), decreased, while 7 lipids increased. In SIT, all 5 lipids, which were free fatty acid (FFA), decreased.

**Conclusion:**

Although both MICT and SIT induced similar and significant improvements in VO_2_peak, serum lipid adaptations to the training differed. The primary changes in serum lipidomic intermediates for both types of training were reductions; however, SIT affected FFA, while MICT predominantly influenced SPs and PLs.

## 1 Introduction

Cardiorespiratory fitness (CRF) among children and adolescents is a concern, as highlighted by a previous study involving 25.4 million individuals aged 6–19 years from 27 countries (Tomkinson and Olds, 2007). CRF is an important marker of cardiovascular health (Raghuveer et al., 2020) and higher level of CRF is related to reduced risks of cardiovascular diseases (Lavie et al., 2015; Keteyian et al., 2008; Al-Mallah et al., 2018; Chu et al., 2020). Cardiovascular disease typically manifests during late adulthood, but evidence suggests that it originates early in life (Mintjens et al., 2018). It is well known that lipoprotein particles and some lipidic intermediates are major contributors to the pathological process of cardiovascular diseases (Soppert et al., 2020). Exercise is one of the effective and cost-efficient strategies to favorably modify cardiovascular risk (Pressler et al., 2020). Both moderate-intensity continuous training (MICT) and sprint interval training (SIT) have been proven effective in improving CRF (Shepherd et al., 2013; Cocks et al., 2016; Scribbans et al., 2014). MICT corresponds to approximately 65%–70% of peak oxygen uptake (VO_2_peak), while SIT is performed at or near 100% VO_2_peak (Gist et al., 2014a; Gist et al., 2014b). The intensity and duration of specific exercises are crucial factors in mediating substrate utilization and intermediate metabolism. Low to moderate-intensity exercises, such as MICT, primarily stress fat oxidation, while high-intensity exercises like SIT place a greater load on carbohydrate metabolism. Although lipids are involved in different exercise-related changes, it is still unclear that the extent and specific types of lipids are involved regarding the exercise intensity, duration and frequency.

Many studies have been conducted on obese adults to investigate the metabolic adaptations induced by different exercise intensities (Keating et al., 2017). Previous studies revealed that acute MICT exerts a more pronounced effect on lipid metabolism than high-intensity interval training (HIIT) in both sedentary (Siopi et al., 2019) and trained male adults (Peake et al., 2014), particularly on serum monounsaturated free fatty acids (FFAs). Studies on the long-term effects of physical exercise on serum lipid metabolism have predominantly focused on serum lipoproteins, such as high-density lipoprotein cholesterol (HDL-C) and low-density lipoprotein cholesterol (LDL-C). Motiani (Motiani et al., 1985) reported that 2 weeks of MICT and SIT could enhance HDL and LDL subclasses, along with increased VO_2_peak, with the improvement after SIT surpassing MICT. Shepherd et al. (2013) observed that 6-week MICT and SIT both effectively enhanced the net breakdown rate of intramuscular triglycerides and comparably improved insulin sensitivity in sedentary adult males. While the long-term MICT and SIT generally yield favorable outcomes for lipid metabolism in adults, their effects on adolescents remain controversial. A total of 14 studies have examined the effects of long-term physical exercise on lipid metabolism in adolescents, with seven of these studies observing significant positive changes, including reductions in LDL-C and enhancements in HDL-C, while the other six reported no significant changes, and one study even observed a negative effect on HDL-C (Stoedefalke, 2007). Therefore, it is speculated that although clinical lipid measures can reflect the benefits of exercise training to a certain degree, it is difficult to detail the variations in the types and quantities of various lipid species.

The lipidome comprises all biomolecules classified as lipids, including those with vast structural diversity and complexity (Merrill et al., 2013). Lipid intermediates regulate various essential functions in normal physiology (Kuo and Hla, 2024). Sphingolipid (SP), a lipid intermediate, is considered an essential constituent of the plasma membrane, where it interacts with cholesterol to form lipid rafts for signal transduction and protein sorting (Chaurasia et al., 2016). Ceramide (Cer), an important SP species involved in the formation of membrane domains (Carreira et al., 2015), can influence the formation and secretion of exosomes (Trajkovic et al., 2008). On the other hand, dysregulation of Cer can affect metabolic functions, and the accumulation of serum Cer levels is associated with the pathogenesis of obesity, insulin resistance, and cardiovascular diseases (Castro et al., 2014). Animal studies have shown that increased Cer levels lead to reduction in insulin action, probably by inhibiting protein kinase B (Hannun and Obeid, 2018). Attenuation of Cer synthesis enhances insulin signaling in diabetes (Kurek et al., 2014) and alleviates obesity-related cardiac dysfunction (Hodson et al., 2015). Some specific serum Cer species are considered as potential indicators of cardiometabolic diseases (Stith et al., 2019; Summers et al., 2019), such as Cer 18:0 and 18:1. Increases in these Cers have been identified as critical predictors of adverse cardiac outcomes in healthy individuals (Havulinna et al., 2016). Fatty acids are not only vital energy substrates for lipid oxidation, but also regulate gene expression, cellular signaling cascades, and arterial function under normal physiological conditions (Yu et al., 2024). However, elevated FFAs in plasma can contribute to obesity, non-alcoholic fatty liver disease, arterial hypertension, and atherosclerosis (Günenc et al., 2022; Savary et al., 2012). Specifically, an excess of saturated FFAs contributes to lipotoxicity (Engin, 2017), which in turn causes the dysfunction and apoptosis of vascular endothelial cells (Raja et al., 2022).

With development in lipidomic techniques combined with bioinformatic analysis, it is now possible to comprehensively study the serum lipidomic profiles in response to different intensities of exercise training. Using targeted lipidomic approach, we previously observed that 6 weeks of SIT on male adolescents induced significant changes in some lipidic intermediates that were strongly associated with alterations in inflammatory markers (Wang et al., 2023). As a continuation of this project, the present study applied MICT and SIT to two groups of male adolescents for 6 weeks to investigate metabolic adaptations in terms of serum lipidomic profiles and lipoproteins. By comparing the effects of these two distinct types of physical exercise training on lipidomic profiles in adolescents, we aimed to provide new insights into the physical exercise features in improving metabolic and cardiovascular fitness in adolescents.

## 2 Materials and methods

### 2.1 Study design

**FIGURE 1:**
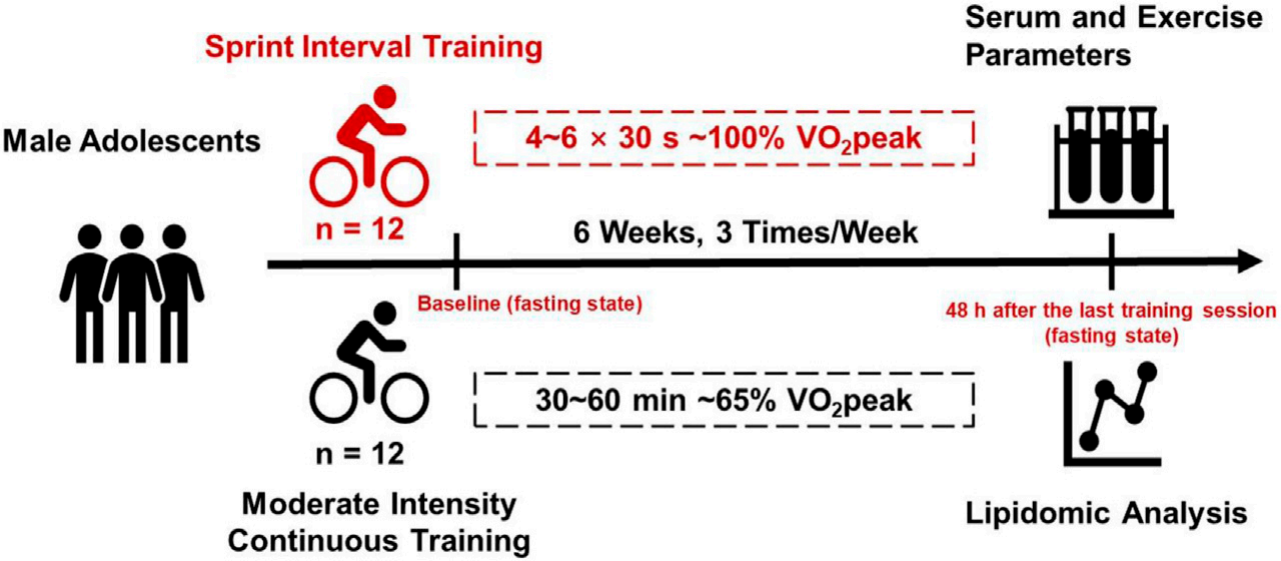
Experimental design of this study. Two groups of male adolescents took part in two different exercise training programs (SIT and MICT) for 6 weeks. Both before and 48 h after the last training session, VO_2_peak was measured and blood samples were collected at fasting state from all the participants and analyzed for lipid metabolism using targeted lipidomic analysis.

Two groups of male middle school students took part in two different exercise training programs for 6 weeks. Both before and 48 h after the last training session, VO_2_peak was measured and blood samples were collected at fasting state from all the participants and analyzed for lipid metabolism using lipidomic analysis. The experimental design was illustrated in Figure 1.

### 2.2 Participants and grouping

Twenty-four healthy male middle school students were recruited through recruitment posters in the campus of a local boarding school. The inclusion criteria were 1) 12–14 years old male adolescents; 2) volunteer to take part in the research; 3) no regular physical training in the past year. Exclusion criteria were 1) history of skeletal muscle/joint injury or pain in the last 3 months; 2) history of exercise-related injuries; 3) chronic disease such as cardiovascular diseases, metabolic diseases, or psychiatric diseases; 4) medicine intake such as anti-inflammation or immunosuppression in the past 3 months. The 24 students were randomly and evenly assigned to either SIT group (n = 12) or MICT group (n = 12) to take part in a specific physical training program for 6 weeks.

Biometric data, including body mass, height, body mass index (BMI), blood samples, and muscle mass were collected from the students both before and 48 h after the last training session at around the same time of the days (8:00–10:00 a.m.). To minimize the impact of an acute training session on circulating metabolites, blood sample was collected 48 h after the completion of the training. The data was collected after overnight fast and refrained from caffeine or any extra nutritional supplementation. VO_2_peak of the students was also measured. All the participants went to the same school and were provided with similar diets; thus, there was no additional dietary control. During the study, the students were instructed to keep their normal dietary habits but refrain from any alcohol or extra physical training. All the twenty-four participants completed all the tests and the whole training process.

The study was approved by the local ethic committee of the Sports Science Experimental Ethics Committee at Beijing Sport University (2020139H) and conducted according to the Declaration of Helsinki. All the participants provided their informed written consent by their p*arents* or guardians, and the privacy of the participants was guaranteed.

### 2.3 VO_2_peak test

VO_2_peak was measured on the Monark 839E (Sweden) using a metabolic gas analysis system (Cortex MetaMax 3B, Cortex, Isernhagen, Germany) for respiration analysis, as described previously (Wang et al., 2023). The participants took a standard warmed-up of 15 min at 25 W, 60 rpm, followed by a 3 min rest. The test started with an initial cycling loading of 25 W, increasing the load by 25 W every 2 min till exhaustion. Test was terminated when: 1) RER>1.1; 2) HR_max_ ≥ 175 b/min; 3) Peak oxygen uptake is maintained for 30s; 4) Rate of perceived exertion (RPE) ≥ 17.

### 2.4 Body composition measurement

A body composition analyzer Inbody 230 (InBody Co., LTD., Seoul South Korea) with bioelectrical impedance analysis technology was used for body composition measurement, including body mass, lean mass, fat mass, and BMI. The measurement was conducted at around 9:00 a.m. in the morning after overnight fasting and reframed from even water.

### 2.5 Blood sample collection

Blood samples were collected via venipuncture through the superficial forearm vein at approximately the same time of the days (8:00 a.m.) and into a tube containing a procoagulant (serum tubes). The samples were coagulated at room temperature for 30 min and then centrifuged at 3,000 rpm and 4°C for 10 min. Serum samples were extracted and frozen at −80°C for further tests. The serum was divided into two parts, one for standard clinical analysis and the other for targeted lipidomic analysis.

### 2.6 Serum lipoprotein profiles

Serum levels of lipoproteins, including total cholesterol, triglyceride, LDL-C, and HDL-C were measured according to routine clinical procedures. Serum concentrations of total cholesterol, triglyceride, LDL-C and HDL-C were determined using biochemical analyzer, Beckman Coulter AU2700 (Brea, CA, United States). Concentration of fasting blood glucose (FBG) was determined using glucose dehydrogenase assay, ACCU-CHEK Active Blood Glucose Meter (Roche Diabetes, Mannheim, Germany). All procedures followed the protocols of manufacturers.

### 2.7 Targeted lipidomic analysis

The procedures of targeted lipidomic analysis referred to our previous study (Wang et al., 2023; Zhang et al., 2023). Initially, lipids were extracted from the serum samples through simple protein precipitation using precooled isopropanol (IPA) at 4°C (Sarafian et al., 2014). Briefly, 95 μL of serum or a pooled quality control sample was spiked with 5 μL of lipid internal standard mixture, followed by the addition of 500 μL of pre-cooled IPA. The mixtures were vortexed for 1 min, placed at −20°C for 10 min, and then vortexed again for another 10 min. After incubating at 4°C for 2 h, the samples were centrifuged at 10,300 × g for 10 min at 4°C, and the supernatants were transferred to a 96-well plate for ultra-high performance liquid chromatography–tandem mass spectrometry analysis.

A semi-quantitative approach was employed using a Waters Iclass-Xevo TQ-S ultra-high-performance liquid chromatography–tandem mass spectrometry system (Waters, Milford, MA, United States) in electrospray ionization mode. Lipid classes were analyzed using a Waters Acquity UPLC BEH Amide column (1.7 μm, 2.1 × 100 mm). Mass spectrometry multi-reaction monitoring method was developed for the confirmation and quantitative evaluation of diverse lipids. Each lipid species had a dwell time of 3 ms. The source nitrogen gas temperature was set to 120°C with a flow rate of 150 L/h. The desolvation gas temperature was maintained at 500°C with a flow rate of 1000 L/h. Capillary voltage was adjusted to 2.8 kV for the positive mode and to 1.9 kV for the negative mode respectively. The autosampler operated at 4°C while the column compartment remained at a constant temperature of 45°C throughout the analysis duration. Solvent A and solvent B consisted of 95% and 50% acetonitrile, respectively, with 10 mM ammonium acetate. The mobile-phase gradient ranged from 0.1% to 20% B for 2 min, followed by an increase from 20% to 80% B for the next 3 min before re-equilibration for an additional 3 min, all at a flow rate of 0.6 mL/min. A volume of 1 μL was injected. System blanks consisting of 95 μL distilled water, 5 μL lipid internal standard mixture, and 500 μL IPA as well as process blanks (IPA) were initially injected thrice each to verify instrument conditions. Quality control samples were injected thrice prior to analyzing biological samples. This process was repeated every 12 samples. Lipidomic raw data processing was performed in TargeLynx in MassLynx v4.1 (Waters, Milford, MA, United States). Each peak was automatically integrated and validated and rectified manually as necessary. The retention time corresponding to internal standards of each lipid class confirmed accurate peak integration for lipids within the same class.

High-performance liquid chromatography (HPLC)-grade acetonitrile, methanol and IPA were purchased from Merck (99.9%; Merck KgaA, Darmstadt, Germany). HPLC-grade ammonium acetate (≤97%) and a lipid internal standard mixture (Avanti Splash Lipidomix™ lipid standards; Avanti Polar Lipids, Inc., Alabaster, AL, United States) containing d7-phosphatidylcholine (15:0/18:1), d7-phosphatidylethanolamine (15:0/18:1), d7-phosphatidylglycerol (15:0/18:1), d7-phosphatidylinositol (15:0/18:1), d7-lysophosphatidylcholines (15:0/18:1), d7-lysophosphatidylethanolamine (15:0/18:1), d7-diacylglycerols (15:0/18:1), d7-TAGs (15:0/18:1), d9-sphingomyelins (18:1), d7-cholesteryl esters (18:1), d7-monoacylglycerols (18:1), and d7-phosphatidic acids (15:0/18:1) were purchased from Sigma-Aldrich (United States).

### 2.8 Training protocol

The training protocols of MICT and SIT referred to a previous study (Macpherson et al., 2011). In brief, the MICT program included continuous cycling workouts on an ergometer (Monark 839E, Sweden) at a workload equal to 65% of VO_2_peak, 3 days a week for 6 successive weeks. For the first 2 weeks, each training session was 30 min, and the third and fourth week, each training session was 45 min, and the last 2 weeks, each training session was 60 min.

The SIT program was consisted of 4-6 bouts of 30-second “all-out” cycling on an ergometer (Monark 884, Sweden) with a resistance of 7.5% of body weight (kg), 4-min resting intervals between two bouts. For the first 2 weeks, each training session included four bouts, the third and fourth week of five bouts, and the last 2 weeks of six bouts. The training frequency per week was the same as MICT.

For both training programs, each training session started with 10 min of warm-up and ended with 10 min cool-down on the ergometer at 60 rpm and 25 W. All training sessions were supervised by professional trainers.

### 2.9 Statistical analysis

Statistical analysis was performed using the SPSS 23.0 (SPSS Inc., Chicago, IL) and GraphPad Prism 8.3.0 (GraphPad Software, LLC) software packages. Anthropometric, biometric data, and serum biochemical measurements were analyzed with two-factor repeated analysis of variance (ANOVA), with group factor (SIT versus MICT) and time factor (pre-training versus post-training) and a significance level set at *p* < 0.05. Tukey’s *post hoc* tests were used to analyze group differences. Generic format data were processed with one-factor statistical analysis using MetaboAnalyst 6.0 (https://www.metaboanalyst.ca/home.xhtml) to conduct principal component analysis (PCA) and generate volcano plots. Lipids with more than 50% missing values were deleted and the KNN (feature-wise) method was used to estimate missing values for the remaining lipids. All lipids were logarithmically transformed (base 10) and automatically scaled (centered on the mean and divided by the standard deviation of each variable) to approximate a normal distribution. Paired-sample *t*-test was used for lipidomic data analysis, with a significance level set at false discovery rate (FDR) adjustment (Benjamini–Hochberg) < 0.05, fold change (FC) of less than 1/1.5 or greater than 1.5, and a 95% confidence interval (CI). Based on the results of significant improvements in VO_2_peak (*p* < 0.001 and partial eta-square of 0.944 in time factor) in both groups, posteriori statistical power was calculated, with a power of 1 by G*Power (version 3.1.9.7) repeated measures ANOVA module. Thus, the sample size of 12 participants in each group was statistically proper.

## 3 Results

### 3.1 Physiological measurements

Results of biometric data and body composition as well as VO_2_peak are presented in Table 1. Two-way repeated measures ANOVA revealed no significant differences in any of the anthropometric parameters at baseline or after the 6-week training between the two groups. However, analysis of VO_2_peak results showed a significant effect of time [F (1, 44) = 14.75, *p* < 0.001], but no significant effect of group [F (1, 44) = 0.20, *p* = 0.65] or interaction between time and group [F (1, 44) = 0.0017, *p* = 0.97]. Tukey’s multiple comparisons test showed that both MICT and SIT resulted in significant improvements in VO_2_peak (from 39.05 ± 8.17 to 47.52 ± 8.51 for MICT and from 40.13 ± 6.37 to 48.42 ± 7.01 for SIT, *p* < 0.05 for both), with no significant difference between the groups.

**TABLE 1:** Biometric data before- and after 6-week physical training.

	MICT		SIT	
	Pre	Post	Pre	Post
Age (year)	13.33 ± 0.89	-	12.83 ± 0.83	-
Height (cm)	164.65 ± 5.25	165.68 ± 5.45	165.28 ± 9.49	166.35 ± 9.42
Weight (kg)	60.85 ± 17.87	61.89 ± 17.72	58.57 ± 13.54	59.80 ± 13.33
BMI (kg/m^2^)	22.20 ± 5.35	22.30 ± 5.15	21.25 ± 3.69	21.43 ± 3.60
Muscle Mass (kg)	25.97 ± 4.55	25.98 ± 5.21	26.28 ± 5.66	27.23 ± 5.41
Fat Mass (kg)	13.50 ± 6.53	13.66 ± 7.68	12.26 ± 4.4	12.34 ± 5.33
VO_2_peak (mL/min/kg)	39.05 ± 8.17	47.52 ± 8.51^*^	40.13 ± 6.37	48.42 ± 7.01^*^

Note: Data are presented as means ± SD (n = 12 per group).

^*^ *p* ≤ 0.05.

### 3.2 Serum lipoprotein profiles

Results of serum lipoprotein analysis are presented in Table 2. Two-way repeated measures ANOVA revealed that none of the parameters including total cholesterol, triglycerides, HDL-C, LDL-C, and FBG, was significantly changed within or between the two groups.

**TABLE 2:** Serum clinical measurements before- and after 6-week MICT and SIT.

	MICT		SIT	
	Pre	Post	Pre	Post
TCHO (mmol/L)	3.79 ± 0.50	3.97 ± 0.50	3.76 ± 0.69	3.87 ± 0.73
HDL-C (mmol/L)	1.49 ± 0.26	1.55 ± 0.28	1.49 ± 0.21	1.57 ± 0.26
LDL-C (mmol/L)	1.97 ± 0.32	2.02 ± 0.25	1.91 ± 0.57	2.05 ± 0.51
TAG (mmol/L)	1.06 ± 0.47	1.07 ± 0.49	0.87 ± 0.26	0.89 ± 0.29
FBG (mmol/L)	5.21 ± 0.36	4.99 ± 0.35	4.83 ± 0.29	5.02 ± 0.44

Note: Data are presented as means ± SD (n = 12 per group). TCHO, total cholesterol; HDL-C, high-density lipoprotein cholesterol; LDL-C, low-density lipoprotein cholesterol; TAG, triacylglycerol; FBG, fasting blood glucose.

### 3.3 Serum lipidomic profiles

**FIGURE 2:**
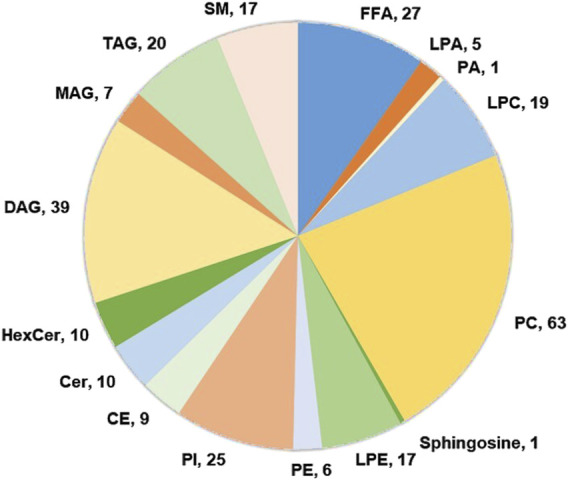
The overall class distribution of lipids measured in serum. FFA, free fatty acyl; LPA, lysophosphatidic acid; PA, phosphatidic acid; LPC, lysophosphatidylcholine; PC, phosphatidylcholine; LPE, lysophosphatidylethanolamine; PE, phosphatidylethanolamine; PI, phosphoinositide, CE, cholesteryl ester; Cer, Ceramide; HexCer, hexosylceramide; MAG, monoacylglycerol; DAG, diacylglycerol; TAG, triacylglycerol, SM, sphingomyelin.

**FIGURE 3:**
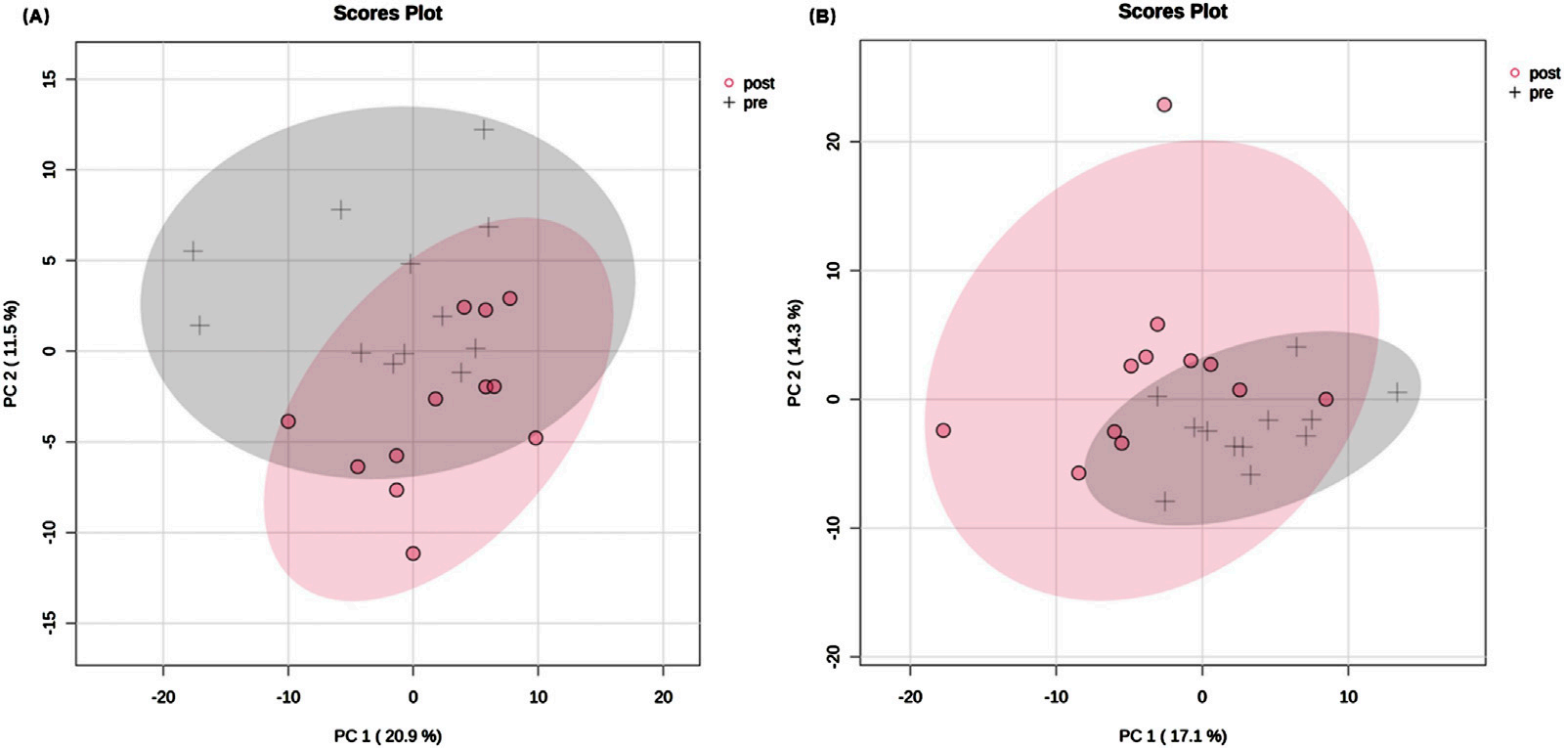
Principal component analysis plots of serum lipids pre- and post-training. **(A)** SIT, **(B)** MICT. In total, 276 lipids were identified in the serum samples of both MICT and SIT. Principal component analysis (PCA) revealed the degree of sample dispersion. PCA on 24 samples and 276 lipids resulted in a model with 2 principal components in each group, which accounted for 31.4% and 32.4% of the total variation after MICT and SIT, respectively. Only the first two principal components and their proportions are displayed in the scores plot. The grey cross represents pre-training sample and the red dot represents post-training sample. The shaded part indicates the 95% confidence interval range.

Using targeted lipidomic analyses, 276 lipids were identified in the serum samples of each group (Supplementary Table S1). The overall class distribution of lipids measured in serum was shown in Figure 2. PCA was conducted on the 276 lipids of each group to detect the degree of dispersion of serum samples taken pre- and post-training of SIT and MICT. For SIT, a model with the first two principal components of 20.9% and 11.5% was revealed, accounting for 32.4% of the total variation (Figure 3A). For MICT, a model with the first two principal components of 17.1% and 14.3% was revealed, accounting for 31.4% of the total variation (Figure 3B). For both SIT and MICT, PCA revealed a partial separation of the serum samples between pre- and post-training.

**FIGURE 4:**
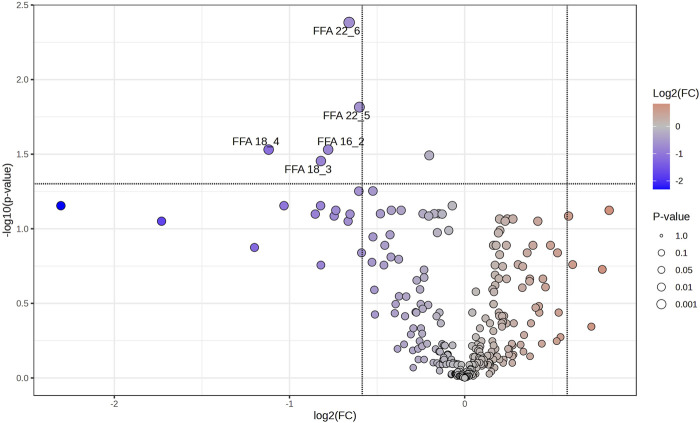
Volcano plot showing the significantly changed serum lipids in SIT. The 6 weeks of training induced significant decrease in 5 serum lipids (blue dots). The volcano plot summarizes both fold-change and *t*-test criteria for all lipids. Lipids with labeling indicate FC < 1/1.5 and FDR <0.05. Specifically, FFA 16:2; 18:3; 18:4; 22:5; 22:6 were decreased.

**FIGURE 5:**
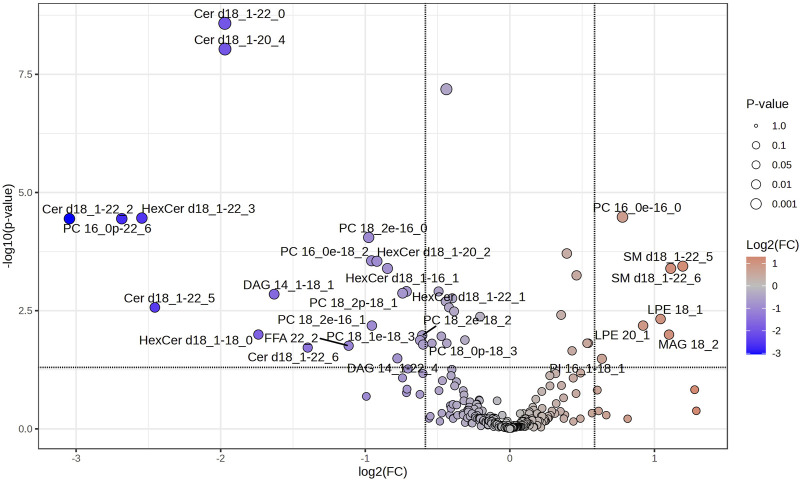
Volcano plot showing the significantly changed serum lipids in MICT. The 6 weeks of training induced significant changes in 28 serum lipids, of which 7 were increased (red dots) and 21 decreased (blue dots). Lipids with labeling indicate FC > 1.5 or <1/1.5, and FDR <0.05. Specifically, SM 18:1–22:5, SM 18:1–22:6, MAG 18:2, LPE 18:1, LPE 20:1, PC 16:0, and PI 16:0–18:0 were increased, while most PC, Cer and HexCer were decreased.

Volcano plots were further conducted to highlight the lipids species altered by the training. Paired-sample *t*-test was applied to the 276 lipids of each group to determine the relative changes of the lipids with significant levels being set at a FC < 1/1.5 or >1.5 (Supplementary Table S2). Among the 276 lipids identified in SIT, 5 lipids (2%) were significantly decreased after training (FDR <0.05, FC < 1/1.5; Figure 4), all of which were FFA (16:2; 18:3; 18:4; 22:5; 22:6). Of the 276 lipids identified in MICT, 28 lipids (10%) were significantly changed, with 7 increased (FDR <0.05, FC > 1.5) and 21 decreased (FDR <0.05, FC < 1/1.5; Figure 5). The increased lipids could be briefly classified into phosphoinositide (PI), phosphatidylcholine (PC) (16:0e-16:0), lysophosphatidylethanolamine (LPE), monoacylglycerol (MAG) (18:2), and sphingomyelin (SM), whereas the decreased lipids were categorized into Cer, hexosylceramide (HexCer), diacylglycerol (DAG), PC (16:0e-18:2; 16:0p-22:6; 18:0p-18:3; 18:1e-18:3; 18:2e-16:0; 18:2e-16:1; 18:2e-18:2; 18:2p-18:1), and FFA (22:2).

**FIGURE 6:**
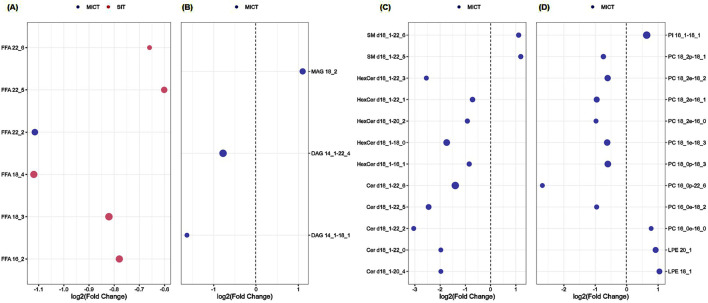
Comparison of the significantly changed serum lipids between MICT and SIT. Of the 28 and 5 lipids in MICT and SIT, respectively, four major lipid species were recognized including FFA **(A)**, glyceride (TG; **(B)**, sphingolipid (SP; **(C)**, and phospholipid (PL; **(D)**. **(A)** All the significantly changed FFAs in both SIT and MICT were decreased, and mostly in SIT (5 FFAs), only one FFA in MICT. **(B)** The significantly changed serum glycerides were only observed in MICT (totally three), of which MAG 18:2 was upregulated, and DAG 14:1–22:4 and 14:1–18:1 were downregulated. **(C)** The significantly changed serum SP was only observed in MICT, and both up- (totally two) and downregulated (totally 10). **(D)** The significantly changed serum phospholipids focused on MICT in which four were increased and eight were decreased. Note: Blue and red dots represent MICT and SIT groups, respectively. The dot size represents FDR-adjusted *p* value.

The significantly changed lipids in MICT and SIT were compared between the two groups (Figure 6). Of the 28 and 5 differential lipids in MICT and SIT, respectively, four major lipid species were recognized: FFA, glyceride (TG), sphingolipid, and phospholipid (PL). In SIT, all five differential lipids were FFA and all showed a decrease (Figure 6A). In MICT, the differential lipids were observed across all four categories, with the majority (24 out of 28) falling into the SP and PL categories (Figures 6C,D), where most (18 out of 24) showed a decrease. Furthermore, one lipid in the category of FFA was also decreased. In MICT, a total of seven lipids were increased, including MAG 18:2, SM d18:1–22:5, SM d18:1–22:6, PI 16:1–18:1, PC 16:0e-16:0, LPE 20:1, and LPE 18:1 (Figures 6B–D). Notably, none of the seven increased lipids in MICT belonged to the FFA category. In contrast, in SIT, all the decreased lipids were FFAs (Figure 6A), whereas in MICT, most of the decreased lipids were from the SP and PL categories (Figures 6C,D).

## 4 Discussion

The study for the first time compared serum lipidomic profiles after 6-week MICT with those of SIT and the results showed significant difference in serum lipid metabolism, despite both training inducing significant but similar improvement in VO_2_peak. In general, both training induced significant reductions in serum lipids. Specifically, SIT had the greatest impact on FFA, while MICT primarily affected SP and PL.

### 4.1 Adaptations in aerobic capacity and serum lipoproteins

The present study reveals that both SIT and MICT markedly improved VO_2_peak, without any significant difference between the groups. Several meta-analyses have clearly demonstrated that VO_2_peak can be improved through continuous aerobic exercise at moderate intensities, as well as high-intensity interval training performed at workloads below or above VO_2_peak. The enhancement of VO_2_peak can be also influenced by various factors, such as an individual’s initial VO_2_peak level, the duration and frequency of training sessions, and genetic factors (Gist et al., 2014c; Milanović et al., 2015; Wen et al., 2019).

The study revealed that none of the serum lipoproteins presented significant change in either SIT or MICT, nor significant difference in any parameter between the two groups. The findings align with previous results showing a significant increase in VO_2_peak but no significant change in serum lipid in overweight/obese men after a 6-week MICT and SIT (Petrick et al., 2021). As suggested, this might be due to the 6-week training period being too short to induce detectable changes in lipid metabolism (Mc et al., 2023). Anyhow, as all the adolescents in the study were healthy with normal serum lipid levels (Elkins et al., 2019), we could not expect the 6-week physical training to further improve or change these parameters. Given that the training programs are different and the adolescents are undergoing continuous growth and development with considerable variations in hormone levels (Chycki et al., 2019), the conventional clinical serum testing method is obviously not sensitive enough to detect the variations in serum lipoprotein adaptation to the different trainings.

### 4.2 SIT and adaptations in serum lipids

The 6-week SIT induced a significant reduction in 5 FFAs, which could be further classified as polyunsaturated fatty acid (PUFA). Physical training, especially endurance exercise, has been shown to enhance the ability of skeletal muscles to oxidize fatty acids, including medium- and long-chain fatty acids, for energy (Van Zyl et al., 1985; Holloszy and Coyle, 1984). Similarly, improved fat oxidation capacity has also been observed after long-term SIT (Bagley et al., 2016). Therefore, the significant reduction in serum FFAs might be attributed to enhanced utilization of FFAs in skeletal muscles.

The effects of physical training on PUFAs are less extensively studied compared to saturated fatty acids (SFAs) and monounsaturated fatty acids (MUFAs). Generally, studies have suggested that increased circulating SFAs and MUFAs, along with decreased PUFAs, were associated with unfavorable cardiometabolic outcomes in adults (Borges et al., 2020; Kaikkonen et al., 2021; Xiao et al., 2024; Jin et al., 2023). However, high serum levels of some specific FAs have also been linked to adverse health risks. For example, γ-linolenic acid (FFA 18:3) has been positively associated with obesity (Kaikkonen et al., 2021). Since physical training generally improves overall lipid metabolism and insulin sensitivity (Jeukendrup, 2010), and the serum lipidomic profile reflects the status of lipid metabolism, the significant reductions in serum PUFAs observed in this study were most likely due to the efficient utilization of these PUFAs.

### 4.3 MICT and adaptations in serum lipids

In MICT, a majority of significantly changed serum lipids were decreased (21 out of 28), particularly in the categories of SP (10 total) and PL (8 total). The ten serum lipids, belonging to SP, could be classified further into two categories: ceramides (Cers; totally 5) and hexosylceramides (HexCers; totally 5). Both Cer and HexCer have been shown to affect cell-signaling and metabolic pathways related with insulin resistance, hepatic steatosis, and major adverse cardiac events (Torretta et al., 2019).

The present study for the first time observed that MICT induced significant reduction in serum Cer levels (totally five Cers) in healthy adolescents. Similar results, however, have been observed in serum of obese and diabetic persons (Bergman et al., 2015; Kasumov et al., 2015) and in muscles of obese male humans (Shepherd et al., 2017) and mice (Mardare et al., 2016) after moderate-intensity exercise training. Cers are composed of sphingosine and a fatty acid and have multifunctional roles in many crucial cellular pathways, such as inflammatory processes and apoptosis (Gaggini et al., 2021). In addition, metabolites involved in ceramide metabolism were negatively associated with VO_2_peak, fasting glucose concentrations and total LDLs, and VLDL cholesterol (Johnson Lawrence et al., 2018).

Five HexCers were significantly decreased in MICT. Currently, there is a lack of research on the impact of physical activity on serum HexCer levels. Nonetheless, hexosylceramides have been reported to be potential biomarkers for the diagnosis of epilepticus (Dickens et al., 2023), colorectal cancer (Elmallah et al., 2022) and chronic multiple sclerosis lesions (Podbielska et al., 2020). Moreover, higher level of HexCer d18:1–20:1 was significantly associated with elevated incidence of type 2 diabetes, probably mediated through β-cell dysfunction (Yun et al., 2020).

In MICT, eight serum lipids belonging to the PL category were significantly decreased, all of which were classified as phosphatidylcholine (PC). PC is the most abundant glycerophospholipids and comprises 40%–50% of total cellular phospholipids (van der Veen et al., 2017). PC plays a crucial role in various biological functions, including cell signaling, lipid metabolism, and the maintenance of cell membrane integrity (Cole et al., 2012). Circulating PC level has been suggested to be associated with running performance (Høeg et al., 2020), and PC supplementation might be beneficial for endurance athletes (Jäger et al., 2007; von Allwörden et al., 1993). Evidence has shown that both acute exercise and long-term exercise training could result in reduction in overall PC levels in muscle (Chorell et al., 2021) and serum (Lin et al., 2023; Beckner et al., 2023); yet compared with untrained persons, well-trained persons generally had relatively higher serum PCs levels (Pikó et al., 2021; Carayol et al., 2017).

PI forms a minor component of the cell membrane compared with other phosphatidylglycerides and is essential for diverse cellular processes, including signaling transduction, membrane dynamics and cellular trafficking (Phan et al., 2019; Viaud et al., 2016). In this study, only one PI, 16:1–18:1, was significantly increased in MICT. Currently, there are no reports on the effects of physical exercise on serum PI level; however, serum levels of PI have been reported to be significantly lower in obese-than in non-obese adults (Yin et al., 2021). Therefore, the increased PI 16:1–18:1 might reflect the beneficial effects of long-term MICT.

## 5 Limitations

It should be noted that the adolescents undergo critical periods of growth and development, which may compound the interpretation of the results based only on blood samples, rather than tissues. Given that serum circulating factors may directly regulate complex processes such as metabolism and the development of chronic diseases, our findings obtained from the blood samples reveal the dissimilar intensities of exercise-induced changes in lipidic metabolites. However, the deep physiological phenotyping of lipidomic still needs further research. Due to the high cost of the lipidomic analyses, the sample size was limited. Despite that we carefully controlled confounders such as diet, stress, sleep patterns, and study environment, future studies with nutrition and food intake tracking might be helpful to draw a more convincing conclusion.

Regarding the lipid class quantification, we acknowledge that there was potential technical limitation when quantitating the PI in the negative ion mode based on fatty acyl transitions, which may lead to potential distortion in summed PI abundances. This limitation may apply to other phospholipid classes quantification. Thus, it needs to be cautious in interpreting the data and comparing the data when different methodology is applied.

The results of the study are expected to provide potential guidelines for healthcare organizations in developing recommendations for adolescents. Additionally, the findings may offer insights into the characteristics of physical training that improve physical fitness in adolescents. However, as this study focused solely on male adolescents, future research on female adolescents is clearly necessary.

## 6 Conclusion

In summary, 6 weeks of MICT and SIT significantly improved aerobic capacity to a similar extent in adolescents. Although conventional serum clinical analysis showed no significant difference in any parameter such as total cholesterol, HDL-C, LDL-C, or triglycerides between the two groups, targeted lipidomic analysis indeed revealed significant differences in serum lipidomic profiles between SIT and MICT. Specifically, while both types of training predominantly reduced serum lipids, MICT induced changes in a broader range of lipid species, including HexCer, Cer, and PC, whereas SIT primarily affected FFA.

## Data Availability

The original contributions presented in the study are included in the article/Supplementary Material, further inquiries can be directed to the corresponding authors.
