# Supplementary material for: Distinct lipidomic profiles but similar improvements in aerobic capacity following sprint interval training versus moderate-intensity continuous training in male adolescents
Source: Front Physiol. 2025 Jan 30;16:1475391. doi: 10.3389/fphys.2025.1475391 (PMC11821953; doi:10.3389/fphys.2025.1475391)
Supplement: Supplementary file 1 [file Table2.docx]

Supplementary Material

# Table S1. 276 lipids identified in the serum samples of MICT and SIT groups.

# Table S2. Fold change of differential serum lipids pre- and post-MICT and SIT with false discovery rate (FDR) adjusted.

| MICT | | | | SIT | | | |
| --- | --- | --- | --- | --- | --- | --- | --- |
|  | FC | log2(FC) | p.ajusted |  | FC | log2(FC) | p.ajusted |
| Cer d18_1-22_2 | 0.12107 | -3.0461 | 3.586E-05 | FFA 22_6 | 0.63304 | -0.65962 | 0.0041434 |
| PC 16_0p-22_6 | 0.15561 | -2.684 | 3.586E-05 | FFA 22_5 | 0.65916 | -0.6013 | 0.015299 |
| HexCer d18_1-22_3 | 0.17135 | -2.545 | 3.468E-05 | FFA 18_4 | 0.46035 | -1.1192 | 0.029475 |
| Cer d18_1-22_5 | 0.18235 | -2.4552 | 0.0026978 | FFA 16_2 | 0.58255 | -0.77954 | 0.029475 |
| Cer d18_1-22_0 | 0.255 | -1.9714 | 2.637E-09 | FFA 18_3 | 0.5661 | -0.82088 | 0.035153 |
| Cer d18_1-20_4 | 0.2552 | -1.9703 | 9.231E-09 |  |  |  |  |
| HexCer d18_1-18_0 | 0.29952 | -1.7393 | 0.0101 |  |  |  |  |
| DAG 14_1-18_1 | 0.32304 | -1.6302 | 0.001417 |  |  |  |  |
| Cer d18_1-22_6 | 0.37943 | -1.3981 | 0.019067 |  |  |  |  |
| FFA 22_2 | 0.46167 | -1.1151 | 0.017465 |  |  |  |  |
| PC 18_2e-16_0 | 0.5082 | -0.97652 | 8.917E-05 |  |  |  |  |
| PC 16_0e-18_2 | 0.51531 | -0.95648 | 0.0002783 |  |  |  |  |
| PC 18_2e-16_1 | 0.51604 | -0.95444 | 0.0065371 |  |  |  |  |
| HexCer d18_1-20_2 | 0.52874 | -0.91937 | 0.0002845 |  |  |  |  |
| HexCer d18_1-16_1 | 0.55617 | -0.8464 | 0.0004044 |  |  |  |  |
| DAG 14_1-22_4 | 0.58317 | -0.778 | 0.032264 |  |  |  |  |
| PC 18_2p-18_1 | 0.59798 | -0.74182 | 0.0013505 |  |  |  |  |
| HexCer d18_1-22_1 | 0.60912 | -0.71521 | 0.0012389 |  |  |  |  |
| PC 18_1e-18_3 | 0.65033 | -0.62075 | 0.013387 |  |  |  |  |
| PC 18_2e-18_2 | 0.65687 | -0.60632 | 0.010556 |  |  |  |  |
| PC 18_0p-18_3 | 0.65985 | -0.5998 | 0.01648 |  |  |  |  |
| PI 16_1-18_1 | 1.5543 | 0.63624 | 0.032936 |  |  |  |  |
| PC 16_0e-16_0 | 1.7158 | 0.77892 | 3.296E-05 |  |  |  |  |
| LPE 20_1 | 1.894 | 0.92143 | 0.0065371 |  |  |  |  |
| LPE 18_1 | 2.0592 | 1.0421 | 0.0047399 |  |  |  |  |
| MAG 18_2 | 2.1446 | 1.1007 | 0.010144 |  |  |  |  |
| SM d18_1-22_6 | 2.1623 | 1.1126 | 0.0004044 |  |  |  |  |
| SM d18_1-22_5 | 2.2907 | 1.1958 | 0.0003612 |  |  |  |  |
